# Physiological Responses of *Crotalaria* spp. to the Presence of High Aluminum Availability in the Soil

**DOI:** 10.3390/plants13162292

**Published:** 2024-08-17

**Authors:** Beatriz Silvério dos Santos, Tassia Caroline Ferreira, Maiara Luzia Grigoli Olívio, Lucas Anjos de Souza, Liliane Santos de Camargos

**Affiliations:** 1 Plant Metabolism Physiology Laboratory, Department of Biology and Zootechny, School of Engineering, São Paulo State University (UNESP), Rua Monção, 226, Zona Norte, Ilha Solteira 15385-000, SP, Brazil; beatriz.silverio-santos@unesp.br (B.S.d.S.); tassia.ferreira@unesp.br (T.C.F.); maiara.olivio@unesp.br (M.L.G.O.); 2 Instituto Federal Goiano, Campus Rio Verde, Rio Verde 75901-970, GO, Brazil; lucas.anjos@ifgoiano.edu.br

**Keywords:** nitrogen metabolism, biological fixation, liming, potentially toxic element, Fabaceae, leguminosae

## Abstract

Brazilian soils are predominantly rich in aluminum, which becomes mobile at pH < 5, affecting sensitive plants; however, some species have developed aluminum tolerance mechanisms. The purpose of this study was to compare the physiological responses of *Crotalaria* genus species, family Fabaceae, which have the ability to associate with nitrogen-fixing bacteria under the influence of Al^3+^ in the soil. The soil used was Oxisol; the experimental design was in randomized blocks in a factorial scheme (2 × 3): soil factor (available toxic aluminum content; correction of dolomitic limestone—MgCO_3_) and species factor (*C. juncea*; *C. spectabilis*; *C. ochroleuca*); cultivated within 43, 53, and 53 days, respectively, with five replications; 30 experimental samples. Mass and length, pigments, gas exchange, and changes in nitrogen metabolism were evaluated. *C. juncea* showed a higher concentration of amino acids in the leaves, internal carbon, and stomatal conductance in soil with Al^3+^, as well as higher production of ureides, allantoinic acid, allantoic acid, proteins, and amino acids in the nodules, with 78% of the Al^3+^ accumulation occurring in the roots. *C. ochroleuca* demonstrated greater shoot length and nodule number production in limed soil; in soil with Al^3+^, it showed a 91% increase in chlorophyll a content and 93% in carotenoids. *C. spectabilis* showed a 93% increase in ureide production in the leaves in soil with Al^3+^.

## 1. Introduction

Aluminum (Al) is the third most abundant metal on the Earth’s surface; it is a constituent element of mineral soil [1]. The most common forms of aluminum found are aluminum oxides and aluminosilicates in stable conditions; among the molecular variations, it has two forms that are considered rhizotoxic, namely Al^3+^ and Al (OH)^4−^ [2]. In acidic soils (pH < 5), Al suffers hydrolysis, which results in free ions dissolved in the water ([Al (H_2_O)_6_]^3+^, referred to as toxic aluminum (Al^3+^) [3]. This form is considered the most toxic to plants, aggressively affecting their root growth and, consequently, the absorption of water and nutrients [4].

Al ions are highly reactive cations; they have a preferential affinity for electron-donating groups, such as carboxyl and phosphates [1]. This element has many potential binding sites that can be harmed, including the cell wall, the plasma membrane surface, the cytoskeleton, and the nucleus [5]. Thus, the organ most damaged by aluminum toxicity is the root system. Al accumulation causes the inhibition of cell elongation at the root apex (cap, meristem, and elongation zone) [6,7], and cell division also occurs in this region; being sensitive to Al, the division is interrupted, resulting in physical damage, such as brittle and stunted roots [5]. Although it causes toxicity to plants, some species have developed different physiological defense mechanisms that promote exclusion or tolerance to aluminum, which can be characterized into two groups, one of which is exclusion that prevents the entry of Al into the root apex (apoplast and symplast), and tolerance to Al, where Al is absorbed and internalized by the plant, resulting in a detoxification process [8].

Advances in research related to agricultural species, both tolerant and sensitive to excess Al in the soil, represent a perspective to reduce the negative impacts that affect crop productivity. Considering that Cerrado is a biome of extreme importance for agriculture, given the cultivation of species such as soybeans (*Glycine max*), corn (*Zea mays*), beans (*Phaseolus vulgaris*), and sugar cane (*Saccharum officinarum*) [9]. However, to maintain productivity without expanding agricultural frontiers, it is necessary to understand the use of new management methods in order to promote sustainable agriculture. Therefore, research focused on species used as green manure adapted to Cerrado stands out as an attractive and viable possibility to address this issue. This would possibly result in a reduction in spending on corrective methods. 

Legumes are primarily used in green manuring due to their nitrogen-fixing capacity, reducing the need for nitrogen-based fertilizers [10,11]. The region where these species are cultivated generally has acidic soils, and understanding the physiological mechanisms involved in determining tolerance and identifying the damage region caused by aluminum can legitimize the recommendation of using Crotalaria species for cultivation in soils with high aluminum concentration.

*Crotalaria Linnaeus* (Fabaceae) comprises about 700 species distributed in tropical and subtropical regions [12,13]. *Crotalaria juncea* aids in soil nutrient cycling and can accumulate potentially toxic metals such as lead [14], nickel [15], and cadmium [16]. This species is highly recommended for crop rotation [17]. *Crotalaria spectabilis* has been implemented in Cerrado soils due to its high adaptability to different tropical locations; its high production of dry leaf mass contributes to soil enrichment through decomposition, and it is also considered a phytoremediation species for toxic metals such as lead [18]. *Crotalaria ochroleuca*, besides being used in green manuring, is known for its potential aluminum tolerance, making it suitable for acidic soils [19].

In choosing the *Crotalaria* species for this study, several factors were considered, especially the established use of these plants in green manuring and their potential benefits in improving soil health. It is expected that *C. juncea*, *C. ochroleuca*, and *C. spectabilis* will exhibit different responses to aluminum stress. *C. juncea* and *C. ochroleuca* are anticipated to show moderate to high tolerance with good biomass production, while *C. spectabilis*, due to its aluminum tolerance and adaptability, is expected to thrive in acidic soils [19]. 

Despite the favorable characteristics of species in this genus, there are few studies on their ability to accumulate potentially toxic aluminum in their biomass and no reports evaluating biometric and physiological characteristics. The novelty of this study lies in demonstrating different responses to high levels of available aluminum so that the potential of the species for the recovery of degraded areas or as green manure in crop rotation and silvopastoral areas can be evaluated.

The hypothesis of the present study is that the presence of toxic aluminum in the species will exhibit decreased changes in gas exchange, nitrogen, and pigment metabolism but will maintain the growth of the root system and shoot part in maintaining the potential for biomass accumulation and growth. Thus, the purpose of the study was to evaluate and compare the species *C. juncea*, *C. ochroleuca*, and *C. spectabilis* from the characterization of physiological responses and biomass accumulation in soil with high aluminum content.

## 2. Material and Methods

### 2.1. Installation of the Experiment and Soil Preparation

The experiment was conducted between March and May 2023 in a controlled greenhouse, with average temperature of 27 ± 2 °C and automatic irrigation (suspended microsprinklers), at UNESP/Campus Ilha Solteira, São Paulo—Brazil (20°43′09″ S 51°33′79″ W). 

The soil used in the experiment was the Oxisol [20] collected in the experimental area of the Farm of Teaching, Research and Extension (FEPE) of the area of cattle culture, Selvíria/MS—Brazil (20°22′11″ S 51°25′9″ W), with depth of 0–40 cm; composite samples were taken for the chemical characterization of the soil [21], 2001); and the textural characterization of the clay soil and total sand and silt, respectively [22] (Table 1).

In order to obtain a soil condition without available aluminum, the calculation was performed for liming correction using the base saturation method [23]. In this method, liming is determined to increase base saturation (V%) to desired values according to each crop. According to the soil analysis, the need for liming (NC) was 1.97 t/ha of dolomitic limestone; the calculation to determine the amount of limestone for liming was made using the following formula:

$$NC=\left(V2-V1\right)\times \frac{T}{PRNT}$$

NC = required amount of limestone per hectare;
T = cation exchange capacity of the soil at pH 7.00;
V2 = base saturation percentage (%), required for the crop in question (V2 = 60%);
V1 = current base saturation percentage (%) of the soil.

The soil was sieved and stored in plastic bags. To incorporate the limestone into the soil, it was mixed with water and incubated for 30 days. To determine the ideal water volume for incorporation, the soil’s water holding capacity was assessed by evaluating the moisture at field capacity (FC). The calculation used to determine FC [24] was as follows: FC = (WS/VF × 100%), where FC = field capacity of the soil, as a % of the volume of soil used; WS = volume of water collected in the graduated cylinder (mL); and VF = volume of soil in the funnel = 100 mL. Therefore, the soil used had a field capacity (FC) of 51%, meaning that for each liter of soil, 0.51 L of water was used, and for incorporation, they were mixed and homogenized manually.

After the incubation period, the soil was transferred to polypropylene pots with a capacity of four liters. Each pot was fertilized with nitrogen, phosphorus, and potassium (NPK) in the 04-14-08 formula; zinc sulfate; and boric acid in the amounts of 0.08, 0.02, 0.01 g/dm³, respectively.

One week after fertilization, new composite soil samples were collected for chemical (Table 1) and physical characterization of the soil (Table 2).

### 2.2. Plant Material and Seed Treatment

The *Crotalaria* seeds were obtained commercially (BRSEEDS^®,^ Araçatuba, Brazil) and subsequently inoculated with *Bradyrhizobium* spp. for all treatments, including the control, to facilitate nodule formation. Following this process, six seeds were placed in each pot, and after the seedlings emerged, thinning was carried out to maintain two plants per pot.

### 2.3. Experimental Design

**Figure 1:**
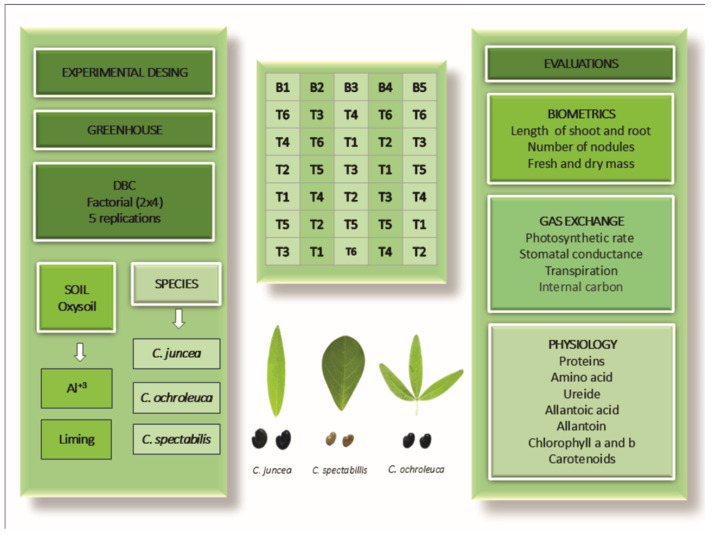
Scheme of design and experimental design in a factorial layout with the distribution of each treatment (Diagram): T1 = *C. juncea* with aluminum, T2 = *C. juncea* with liming, T3 = *C. spectabilis* with aluminum, T4 = *C. spectabilis* with liming, T5 = *C. ochroleuca* with aluminum, and T6 = *C. ochroleuca* with liming and types of evaluations (biometrics, gas exchange, and physiology).

The experiment was organized in randomized blocks, using a factorial design (2 × 3), with 5 biological replicates per treatment, totaling 30 biological samples. The first factor evaluated two distinct soil types: one with a high level of available toxic aluminum and the other amended with dolomitic lime (MgCO_3_). The second factor consisted of three species of the genus *Crotalaria*: *C. juncea, C. spectabilis,* and *C. ochroleuca*. Each species was, thus, subjected to two soil conditions, one with available aluminum and the other with liming, with five replicates for each treatment (Figure 1).

### 2.4. Gas Exchange

Gas exchange analysis was performed on the plants at 30 days to determine their photosynthetic responses using the portable gas exchange analysis device, CIRAS-3 (Portable Photosynthesis System—PP System), adapted to specific environmental conditions. Measurements were taken on two leaves from each repetition, with the selected leaf positions being at the 3rd or 4th node from the apical meristem, which was fully developed and undamaged, between 10:00 a.m. and 12:00 p.m.

For the leaf analysis, they were carefully positioned inside the universal leaf chamber PLC3, which has dimensions of 18 × 25 mm, allowing for the accommodation of two to three leaves simultaneously. The controlled conditions included a standard CO_2_ concentration of 390 µmol/mol and a fixed humidity of 60%. The airflow in the chamber was kept constant at 300 mL/min, while in the analyzer, it was set at 100 mL/min. Ambient light was used for illumination, and an infrared (IR) sensor was employed to measure leaf temperature. The leaf area considered was 4.50 cm^2^, and the boundary layer resistance was calculated as 0.40 m^2^/s/mol. The stomatal ratio was fixed at 50%, ensuring adequate gas exchange. It is important to note that the system used was automatically calibrated, following the procedures described by Bomfim et al. (2023) [25] and Zhang et al. (2018) [26], ensuring the precision of the obtained results.

The data obtained by the equipment were processed using the formulas from [27] Parkinson et al. (1980), which encompass various parameters, including the photosynthetic rate (A, in µmol CO_2_ m^2^/s), stomatal conductance (Gs, in mol H_2_O m^2^/s), transpiration (E, in mmol H_2_O m^2^/s), and internal CO_2_ concentration (Ci, in µmol mol⁻¹) [28].

### 2.5. Collection and Biometric Evaluations

For the collection of species, the phenological stage was considered, prioritizing collection during the vegetative phase, i.e., before the full flowering period. In this context, different species were collected at different times after sowing. *C. juncea*, *C. spectabilis*, and *C. ochroleuca* were collected at 43, 53, and 53 days APS, respectively.

The plant material obtained during collection was separated into leaves, roots, and nodules (if they were formed). Then, it underwent a process of washing with distilled water and drying using paper towels. Fresh mass (g) of nodules, roots, and aerial parts (leaves and stems) was measured using a precision balance. Additionally, root length (cm) and aerial part length were measured using a graduated ruler. Manual counting of nodules was also performed as part of the procedure.

Subsequently, the aerial and root parts of the plant samples were dried in a forced air circulation oven, maintained at a temperature of 65 °C for 72 h. After this procedure, the samples were weighed (g) using an analytical balance to determine the dry mass.

### 2.6. Pigments

With the freshly collected biological material, a portion of the leaves from the aerial part was separated for the extraction and quantification of total chlorophylls [29] and carotenoids [30]. The remaining portion, consisting of the aerial part, roots, and previously identified nodules, was stored in a freezer to allow future extraction and quantification of various compounds present in the material.

In the procedure for extracting chlorophyll and carotenoids, the leaves were cut into thin strips, avoiding the central vein. Subsequently, these strips were weighed (0.050 g) using an analytical balance and then placed in test tubes. A total of 7.00 mL of DMSO (dimethyl sulfoxide) was added to these tubes, which were then subjected to a water bath with a hood, keeping the solution in the dark at a temperature of 65 °C for 35 min. After cooling, still in a dark environment, the quantification readings were taken using a spectrophotometer, employing specific wavelengths of 663 nm and 645 nm for chlorophylls a and b, respectively, and 480 nm for carotenoids. For the measurement of each pigment, the following formulas were used:

$$Chla=\left(12.7\times \left|663\right|\right)-\left(2.69\times \left|645\right|\right);\;Chlb=\left(22.9\times \left|645\right|\right)-\left(4.68\times \left|663\right|\right);$$

### 2.7. Extraction of Soluble Compounds and Quantification

To extract the soluble compounds present in the plant material, the leaves, roots, and nodules were separately weighed (g) using an analytical balance. Each organ was then macerated to extract the compounds, which were quantified individually.

For maceration of the material, MCW was used in the following proportions: for nodules, 0.250 g MF for 2.50 mL of MCW; for leaves, 0.500 g MF for 5.00 mL; and for roots, 0.250 g MF for 3.5 mL of MCW. Subsequently, they were transferred to Falcon tubes for centrifugation to promote separation of the material into two phases: supernatant and precipitate [31]. After phase separation, each was used for different purposes, with the supernatant used for the extraction of total soluble compounds and the precipitated plant material reserved for protein extraction.

To continue the extraction of total soluble compounds, the resulting supernatant was transferred to a graduated Falcon tube, where the volume was recorded to calculate the appropriate proportion of chloroform and water. For every 4.00 mL of supernatant in the Falcon tube, 1.00 mL of chloroform and 1.5 mL of distilled water were added and then stored in the refrigerator for 24 h. After this period, the supernatant liquid separated into two phases, liposoluble and hydrosoluble. The hydrosoluble phase was used as an aliquot for subsequent analyses of amino acids [32] and ureides, namely allantoin and allantoic acid [33], while the liposoluble phase was discarded.

To continue protein extraction, the precipitate from the first extraction was used, then subjected to the addition of NaOH (sodium hydroxide) and subsequently centrifuged for 15 min. The resulting supernatant was used as an aliquot for protein quantification [34].

#### 2.7.1. Ureides: Allantoin and Allantoic Acid

Ureide quantification comprised four distinct steps. In the first phase, an aliquot of the hydrosoluble phase was used, adding it to test tubes along with distilled water, 0.5 N NaOH, and a drop of 0.33% phenylhydrazine. The mixture was then heated in a water bath at 100 °C for eight minutes and cooled to room temperature. In the second step, 0.65 N HCl (hydrochloric acid) was added to the tube, which was sealed, heated in a water bath at 100 °C for 4 min, and cooled to room temperature. In the third step, 0.4 N phosphate buffer, pH 7.0, and 0.33% phenylhydrazine were added, waiting for five minutes at room temperature and five minutes in the freezer. In the fourth step, the test tubes were placed on an ice tray, keeping them refrigerated, and 37% HCl was added, which was previously stored in the refrigerator to ensure its cold temperature at the time of the assay. Then, 1.25% potassium ferricyanide (K_3_Fe (CN)_6_) was added, shaking the tubes with a vortex, removing them from the ice tray, and waiting 15 min at room temperature before reading them on the spectrophotometer at 535 nm.

The procedure for quantitative determination of allantoic acid was similar to the ureide assay, differing only in the first step, in which only the aliquot and distilled water were added. The subsequent steps were performed identically to the method used for ureides.

The determination of allantoin quantity was obtained by subtracting the values corresponding to ureides and allantoic acid.

#### 2.7.2. Proteins

In protein analysis, a portion of the aliquot was placed in a test tube, to which Bradford reagent was added. After waiting for a period of three minutes at room temperature, the reading was performed on the spectrophotometer using a wavelength of 595 nm.

To prepare the Bradford reagent, 200 mg of Coomassie Brilliant Blue G-250 and 50 mL of methanol were used. The mixture was stirred for 2 h, and 100 mL of 85% H_3_PO_4_ was added. After resting for 24 h, the volume was adjusted to 1 L with distilled H_2_O and filtered twice.

#### 2.7.3. Amino Acids

In a test tube, a portion of the aliquot corresponding to the hydrosoluble phase of compound extraction was introduced. Additionally, citrate buffer 0.2 M, pH 5.0, 5% ninhydrin in methyl glycol and KCN (potassium cyanide) 0.0002 M were added. After sealing, the tube was heated in a water bath at 100 °C, maintaining this condition for 20 min. Then, it was cooled to room temperature for ten minutes, followed by the addition of 60% ethanol. Subsequently, the reading was performed on the spectrophotometer at a wavelength of 570 nm.

### 2.8. Aluminum Analysis in Plant Biomass

For aluminum analysis, 1 mL of nitroperchloric digestion and 5 mL of the aluminum complexing solution were pipetted. To prepare the complexing solution, 1% gelatin, buffer solution with pH 5.30 (NH_4_OH and CH_3_COOH), 0.1% aluminon, 1% ascorbic acid (C_6_H_8_O_6_), and calcium chloride (CaCl_2_) 0.1 M were used. After adding the complexing solution, the samples were stirred and placed in a water bath for 15 min at 80 °C. After cooling, the reading was performed on the spectrophotometer at a wavelength of 520 nm. The readings were taken in transmittance, converted to absorbance, and subsequently used to determine the aluminum concentration in the plant extract [35].

### 2.9. Aluminum Accumulation in Dry Biomass, Transfer Factor, and Tolerance Index

The aluminum accumulation (mg/organ) was determined using the dry mass data and aluminum concentration in the respective organs: root, aerial part, and total biomass. To calculate the aluminum accumulation in the root (RAC), the root dry mass (RDW), and the aluminum concentration in the root (RMC), the following formula was used:

$$RAC=\frac{RDW\times RMC}{1000}$$

For the accumulation of aluminum in the aerial part (SAC), the aerial part dry mass (SDW), and the aluminum concentration in the aerial part (SMC), the calculation was as follows:

$$SAC=\frac{SDW\times SMC}{1000}$$

The total aluminum accumulation in the biomass (TAC) was obtained by summing the values of RAC and SAC, being expressed in milligrams per dry organ mass (mg/organ).

These calculations were essential to evaluate the metal’s mobility potential in the plant species, with the translocation index (TI%) as described by Rahman et al. (2013) and the metal’s potential to be transferred from the soil to the plant, with the transfer factor (TF) based on Kabata-Pendias and Pendias (2001). The translocation index (TI%) was expressed as the percentage of aluminum transferred to the aerial part, determined by the following formula:

$$TI\left(\%\right)=\left(\frac{SAC}{TAC}\right)\times 100$$

The transfer factor (TF) was calculated by the ratio between SMC and the initial soil aluminum concentration (before liming and fertilization) (SBMC) using the following formula:

$$TF=\frac{SMC}{SBMC}$$

The tolerance index (TI) was calculated as the ratio between the total dry biomass of the treatment (TBDW) and the total dry biomass of the control (TBDW control):

$$TI=\frac{TBDW}{TBDW\left(control\right)}$$

### 2.10. Statistical Analysis

The normality and homoscedasticity of the data were tested by Shapiro–Wilk (*p* ≤ 0.05) and Levene for Tukey (*p* ≤ 0.05). When a dose–response relationship was indicated to answer the hypothesis, analysis of variance (ANOVA) was used through the F-test (*p* ≤ 0.05), and when significant, the variables were subjected to Tukey’s test (*p* ≤ 0.05).

Statistical analysis and graphing were performed and documented using protocols developed in R software version 4.2.1 [36].

## 3. Results

### 3.1. Gas Exchange 

**Figure 2:**
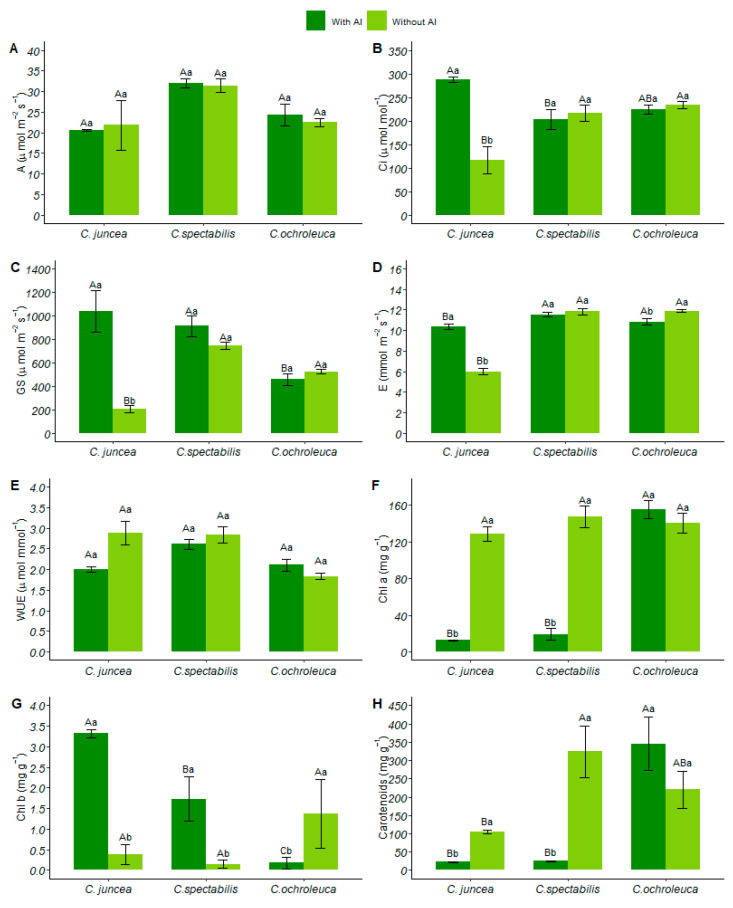
(**A**) Net photosynthesis (µmol CO₂ m²/s), (**B**) internal leaf carbon (µmol mol^−^¹), (**C**) stomatal conductance (mol H₂O m²/s), (**D**) transpiration (mmol H₂O m²/s), (**E**) water use efficiency (µmol CO₂ mmol H₂O^−^¹), (**F**) chlorophyll a quantification (mg·g^−^¹), (**G**) chlorophyll b quantification (mg·g^−^¹), (**H**) carotenoid quantification (mg·g^−^¹). Each species is represented by two columns, with dark green columns indicating soil with available aluminum and light green columns indicating soil with liming. Uppercase letters indicate the comparison of means between different plant species under the same soil conditions, while lowercase letters compare the means between soil conditions for each plant species.

There was no interaction between the factors analyzed for net photosynthesis. However, *C. spectabilis* recorded the highest photosynthetic rate in soil with aluminum (32.00 ± 2.64), surpassing other *Crotalaria* species by 32% (Figure 2A). Table S1 contains the table of analysis of variance of liquid photosynthesis. 

In soil with available aluminum, *C. juncea* presented the highest concentration of internal carbon (287.75 ± 14.37) and had a 30% increase compared to *C. spectabilis*. However, in limed soil, it had the lowest performance (116.51 ± 65.91), presenting a 51% reduction in relation to *C. ochroleuca* (Figure 2B). 

In soil with available aluminum, *C. juncea* increased stomatal conductance by 57% compared to *C. ochroleuca*, which had a lower mean (457.2 ± 120.05). Furthermore, *C. juncea,* in soil conditions with aluminum, showed a 79% increase in stomatal conductance in relation to soil with liming (Figure 2C).

Both in soil with liming and aluminum, *C. juncea* showed a significant decrease in leaf transpiration compared to other *Crotalaria* species, with reductions of 10% and 49%, respectively. However, when analyzing the species individually, a higher transpiration rate was observed in soil with available aluminum (10.34 ± 0.51) than in soil with liming (6.01 ± 0.62) (Figure 2D).

*C. ochroleuca* grown in soil with liming (2.10 ± 0.33) showed a 36% reduction in water use efficiency compared to other species. Similarly, *C. juncea* showed a 30% reduction in water use efficiency when grown in soil with available aluminum (Figure 2E).

### 3.2. Pigments

In soil with the presence of aluminum, *C. ochroleuca* exhibited a significantly higher amount of chlorophyll a compared to the other species (155.18 ± 22.00), representing a 91% increase. Furthermore, when examining the chlorophyll a of *C. ochroleuca* in different soil conditions, there was no aluminum influence on the amount of this pigment (Figure 2F). 

Chlorophyll b content varied between species in soil with aluminum. *C. juncea*, in particular, had a 95% increase. Both *C. juncea* and *C. spectabilis* showed higher levels of this pigment in soil with aluminum, with values of 3.32 ± 0.22 and 1.73 ± 1.2, respectively. However, for *C. ochroleuca* in limed soil, the content was significantly higher, showing an 88% increase compared to the other species (Figure 2G).

For total chlorophyll content, no interaction was observed between the analyzed factors. In soil with aluminum, *C. ochroleuca* showed a concentration 87% higher compared to the other species. Table S2 contains the table of analysis of variance of total chlorophyll. 

*C. ochroleuca* had higher carotenoid content (345.10 ± 162.133), while *C. juncea* and *C. spectabilis* did not show statistical differences in soil with available aluminum. Despite the *C. ochroleuca* having produced 93% more carotenoids compared to other species, it did not differ statistically in the soil conditions, determining that aluminum did not interfere with the production of this pigment for the species in question (Figure 2H).

### 3.3. Biometrics

**Figure 3:**
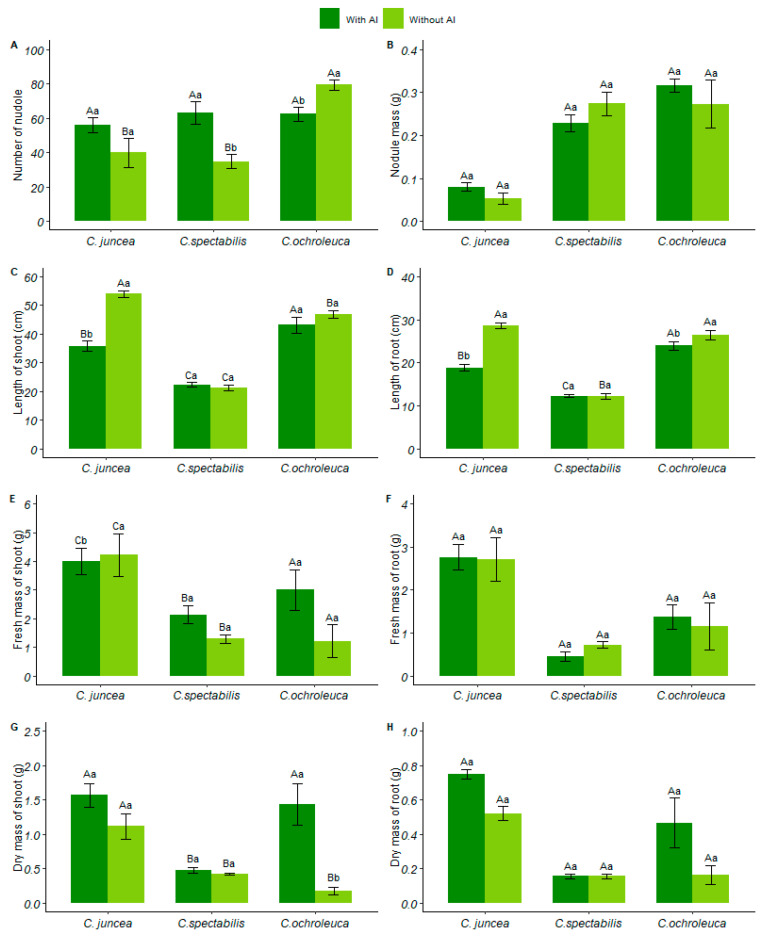
The data refer to the species of Crotalaria: (**A**) number of nodules, (**B**) nodular mass (g/plant), (**C**) length of the aerial part (cm/plant), (**D**) length of the root (cm/plant), (**E**) fresh mass of the aerial part (g/plant), (**F**) fresh mass of the root (g/plant), (**G**) dry mass of shoot (g/plant), (**H**) dry mass of root (g/plant). Each species is represented by two columns, with dark green columns indicating soil with available aluminum and light green columns indicating soil with liming. Uppercase letters indicate the comparison of means between different plant species under the same soil conditions, while lowercase letters compare the means between soil conditions for each plant species.

*C. ochroleuca* species formed more nodules (79.32 ± 6.72) in limed soil, presenting a superior performance compared to the other species. *C. spectabilis* produced 45% more nodules in soil with aluminum than in soil with liming (63.08 ± 14.07) (Figure 3A). Although nodule mass showed no interaction between factors, *C. ochroleuca* nodules increased by 22% compared to other species (Figure 3B). Table S3 contains the table of the analysis of variance of nodule mass. 

**Figure 4:**
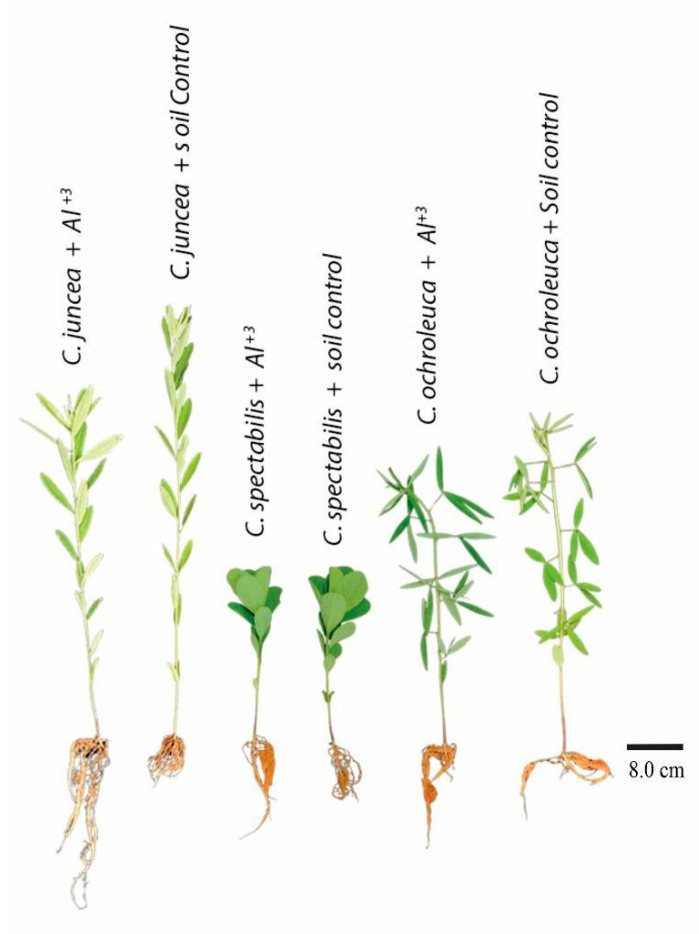
The species *C. juncea* was grown with aluminum and limed soil (soil control) for 43 days. The species *C. ochroleuca* was grown with aluminum and limed soil for 53 days, as was *C. spectabilis*. The plants were randomly selected for the photograph.

In soil treated with liming, *C. juncea* demonstrated a 39% increase in stem growth compared to the other species, presenting a 53.94 ± 2.54 value. In limed soil, aluminum had a positive effect on *C. ochroleuca*, which grew 51% more than the other *Crotalaria* species, reaching a 43.1 ± 6.10 value (Figure 3C and Figure 4).

*C. spectabilis* had the shortest root length compared to the other species, both in soil with aluminum (12.2 ± 0.63) and in soil with liming (12.15 ± 1.34), with reductions of 49% and 58%, respectively. However, when analyzing soil conditions, there was no statistical difference, indicating that root length was not influenced by aluminum content in the soil (Figure 3D and Figure 4).

There was no interaction between the factors for the fresh mass of the shoot. Although there was only a 5% reduction in *C. juncea* fresh mass in the presence of aluminum, this reduction was minimal. Likewise, for the fresh mass of the shoot, *C. juncea* in soil with aluminum showed a 3% increase in relation to the soil with liming (Figure 3E).

Although there were no significant interactions in root fresh mass, *C. juncea* presented a higher mass compared to the other species, around 21% larger. Furthermore, the soil with aluminum stimulated root growth (2.762 ± 0.29). Root masses in soils with aluminum and liming showed no significant differences, indicating that aluminum did not influence the root mass of the species (Figure 3F).

Species *C. juncea* (1.56 ± 0.39) and *C. ochroleuca* (1.43 ± 0.67) showed greater shoot dry mass in soil with aluminum, while *C. spectabilis* (0.48 ± 0.08) had a 30% reduction in relation to *C. juncea*. *C. ochroleuca* presented a higher dry mass in soil with aluminum than in soil with liming, indicating that aluminum positively influenced the dry mass of the shoot (Figure 3G).

For root dry mass, there was no interaction between the factors. Even without significant interactions, we observed that *C. juncea* had the highest root dry biomass (0.75 ± 0.06), with a 30% increase compared to the limed soil (Figure 3H). Table S4 contains the table of analysis of variance of nodule mass.

### 3.4. Quantification of Compounds

#### 3.4.1. Nodules

**Figure 5:**
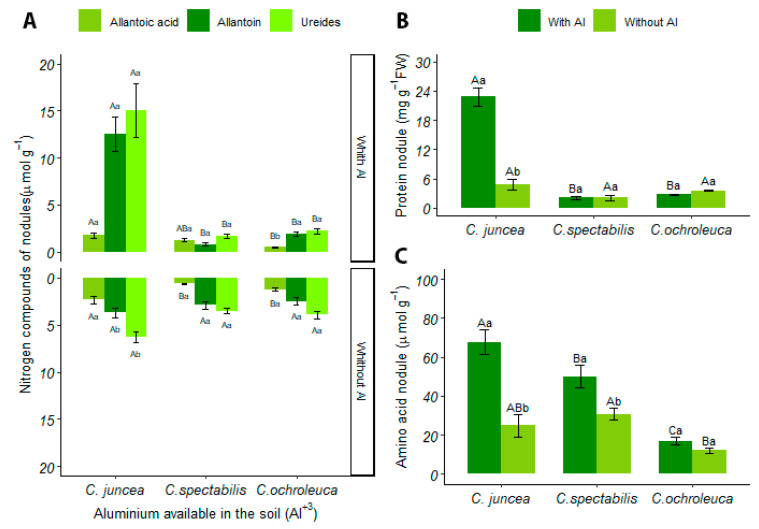
(**A**) Concentration of allantoinic acid, allantoin, and ureides in nodules (µmol·g^−1^), (**B**) total protein concentration in nodules (mg g^−1^ FW), (**C**) concentration of soluble amino acids in nodules (µmol·g^−^¹). Uppercase letters indicate the comparison of means between different plant species under the same soil conditions, while lowercase letters compare the means between soil conditions for each plant species.

*C. juncea* had a higher concentration of ureides in nodules in soil with aluminum, with an 89% increase when compared to other *Crotalaria* species. Furthermore, when considering the soil condition, there was a 58% increase in production in soil with aluminum (15.01 ± 6.40) compared to soil with liming (6.25 ± 1.26) (Figure 5A). 

In limed soil, *C. juncea* produced 74% more allantoic acid in nodules compared to other species. In soil with aluminum, this production was 70%. When observing soil conditions, the only species that showed a statistical difference was *C. ochroleuca*, which showed a higher concentration of allantoic acid in limed soil (1.21 ± 0.47) compared to soil with available aluminum (Figure 5A).

In allantoin production, *C. juncea* presented a higher mean in limed soil compared to the other species, representing a 93% increase in production. However, when observing the soil conditions of each species individually, *C. juncea* obtained a higher production of allantoin in soil with available aluminum compared to soil with liming, with 12.56 ± 4.04 and 3.71 ± 1.08 means, respectively (Figure 5A).

The same was repeated for the production of proteins in the nodules, where *C. juncea* obtained greater production compared to other species, representing an approximate 90% increase. In soil conditions with available aluminum, *C. juncea* also had a higher mean, with 22.75 ± 4.11, compared to the limed soil, which recorded 4.78 ± 2.23 (Figure 5B).

For amino acids in nodules in soil with aluminum, *Crotalaria* species are different from each other, with *C. juncea* presenting the highest production (67.82 ± 14.20), followed by *C. spectabilis* (49.99 ± 12.88) and *C. ochroleuca* (16.99 ± 4.52). Regarding soil conditions, *C. juncea* and *C. spectabilis* showed statistical differences, with higher amino acid production in soils with available aluminum, 63% and 38%, respectively (Figure 5C). 

#### 3.4.2. Root 

There was no interaction between soil factors and *Crotalaria* species in the production of ureides in the root. However, the *C. spectabilis* had a 76% increase in this compound (2.51 ± 0.82). Table S5 contains the table of analysis of the variance of ureids in the root.

**Figure 6:**
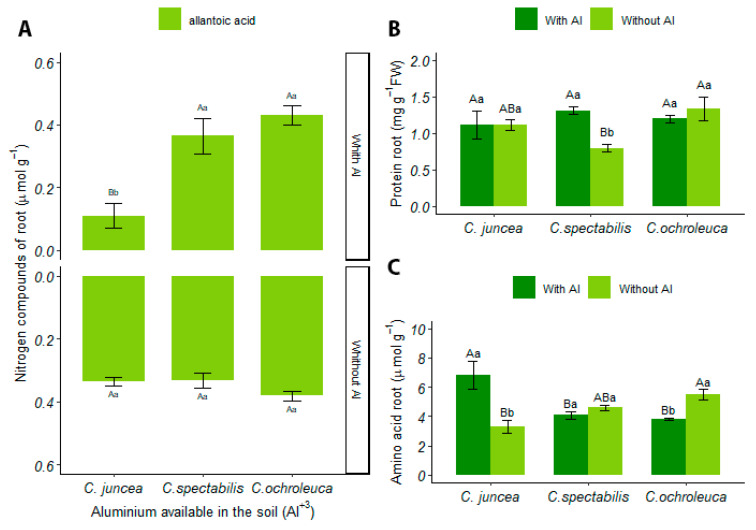
(**A**) Concentration of allantoinic acid in roots (µmol·g^−1^), (**B**) total protein concentration in roots (mg g^−1^ FW), (**C**) concentration of soluble amino acids in roots (µmol·g^−1^). Uppercase letters indicate the comparison of means between different plant species under the same soil conditions, while lowercase letters compare the means between soil conditions for each plant species.

In soils with available aluminum, allantoic acid in the roots of *C. juncea* differed from the other species, reducing by 74% and presenting the lowest mean, with 0.11 ± 0.09. Under soil conditions, there was a greater production of allantoic acid in limed soil, recording a 67% increase compared to soil with available aluminum (Figure 6A).

There was no significant interaction between the factors for the presence of allantoin in the roots. However, it is noteworthy that, in limed soil, the general mean of allantoin production was higher for *C. spectabilis* (1.98 ± 0.77), representing a 52% increase compared to the other species. Table S6 contains the table of analysis of the variance of allantoin in the root. 

The protein content in soil with liming showed that *C. spectabilis* has the lowest mean (0.79 ± 0.10) compared to the other species, presenting a reduction in proteins by 40%. When evaluating the values under soil conditions, this species presents 39% more protein production in the root in soil with aluminum than in soils with liming (Figure 6B).

The protein content in liming soil showed that *C. spectabilis* has the lowest mean (0.79 ± 0.10) compared to the other species, indicating a 40% reduction in the number of proteins. However, when evaluating the values in different soil conditions, it is observed that this species presents a 39% increase in protein production in the root in soil with aluminum compared to that with liming (Figure 6B).

*C. juncea* stands out from the other species by presenting the highest production of amino acids, with around 44% more compared to the others, totaling a mean of 6.83 ± 2.13. Furthermore, its concentration is significantly lower in the limed soil compared to the soil with aluminum, measuring 3.33 ± 0.98, where there is a 51% reduction (Figure 6C).

#### 3.4.3. Leaves

The concentration of ureides in the leaves did not demonstrate a significant interaction between the factors analyzed. However, *C. ochroleuca* showed a 17% higher production of ureides in the leaves compared to the other species, presenting an overall mean of 7.63 ± 0.93. Table S7 contains the table of analysis of the variance of ureides in the leaves.

**Figure 7:**
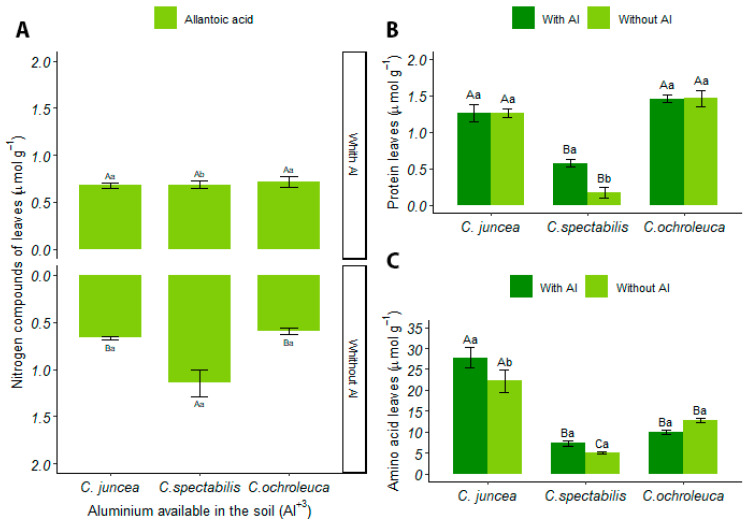
(**A**) Concentration of allantoinic acid in leaves (µmol·g^−^¹), (**B**) total protein concentration in leaves (mg g^−^¹ FW), (**C**) concentration of soluble amino acids in leaves (µmol·g^−^¹). Uppercase letters indicate the comparison of means between different plant species under the same soil conditions, while lowercase letters compare the means between soil conditions for each plant species.

For allantoic acid in limed soils, *C. spectabilis* presented a higher concentration of 1.17 ± 0.317 compared to *C. juncea* and *C. ochroleuca*, with 0.66 ± 0.04 and 0.59 ± 0.07, respectively. Furthermore, *C. spectabilis* differed statistically across soil conditions, where limed soil represented 39% more allantoic acid concentration compared to soil with available aluminum (Figure 7A). 

No significant interaction was observed in the concentration of allantoin in the leaves; however, *C. juncea* in soil with available aluminum had a lower overall mean of allantoin (5.78 ± 0.66) compared to the other species, representing a 14% reduction. Table S8 contains the table of analysis of the variance of allantoin in the leaves. 

In soil with available aluminum, *C. ochroleuca* stood out by presenting the highest protein production in the leaves (1.45 ± 0.12), surpassing the other species by 60%. On the other hand, *C. spectabilis*, in this same type of soil, recorded a 70% increase in protein production compared to the limed soil (Figure 7B).

In amino acid concentration in leaves, *C. juncea* showed a 73% increase compared to *C. spectabilis*. However, in different soil conditions, *C. juncea* presented a higher mean for amino acid production in soil with aluminum than in soil with liming, with values of 27.75 ± 5.52 and 22.24 ± 5.98, respectively (Figure 7C).

### 3.5. Aluminum Accumulation in Biomass and Tolerance Index 

*Crotalaria* species demonstrate tolerance to the aluminum content present in the soil despite a 36% reduction in the index in soil with aluminum. Although a significant interaction between the factors was not observed, both for the accumulation of metal in the roots and shoot, the species *C. juncea* stood out among the *Crotalaria* species as the one that accumulated the most aluminum in the biomass, showing an increase in 78% in the roots and 68% in the shoot. This resulted in a total aluminum accumulation in biomass of 78% relative to other *Crotalaria* species. 

*C. juncea* was the species that transferred the most aluminum from the soil to the plant, whether in soil with aluminum or in soil with liming, with 88% and 72%, respectively, when compared to the other species. *C. juncea* transferred more aluminum from the soil to the plant, around 53% more than in limed soil. 

The species *C. juncea* was the most efficient in transferring aluminum from the soil to the plant, whether in soil with or without liming, recording rates of 88% and 72%, respectively, surpassing the other species. Notably, in limed soil, *C. juncea* absorbed 53% more aluminum from the soil into the plant compared to the other species.

**Figure 8:**
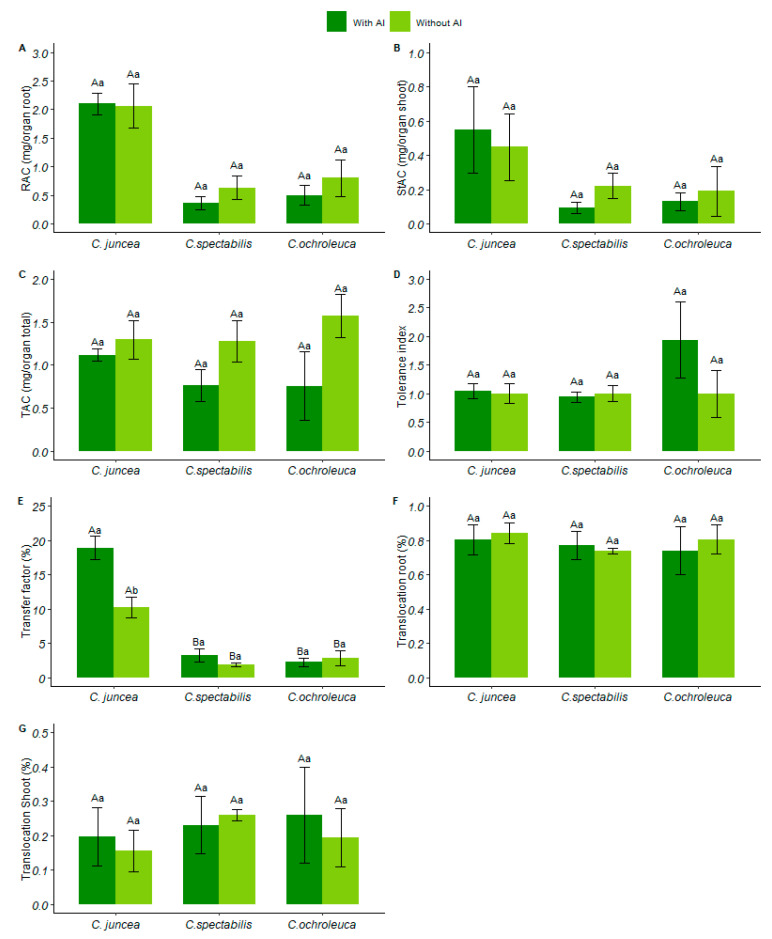
(**A**) Root accumulation (mg/organ root), (**B**) shoot accumulation (mg/organ shoot), (**C**) total accumulation (mg/organ total), (**D**) tolerance index, (**E**) transfer factor (%), (**F**) root translocation index (%), (**G**) shoot translocation index (%). Each species is represented by two columns, with dark green columns indicating soil with available aluminum and light green columns indicating soil with liming. Uppercase letters indicate the comparison of means between different plant species under the same soil conditions, while lowercase letters compare the means between soil conditions for each plant species.

Aluminum translocation within plants, especially in roots, was most evident in *C. juncea*, with a 6% increase compared to other *Crotalaria* species (0.84 ± 0.105). On the other hand, in the root-to-the-shoot movement, *C. spectabilis* showed an 11% increase in relation to the other species (See Figure 8).

## 4. Discussion

### 4.1. Aluminum Influence on the Photosynthetic Process and Plant Growth 

The presence of excess Al in the soil can have harmful effects on plant physiology, inhibiting root growth; interfering with the absorption of nutrients such as phosphorus, calcium, and magnesium; and inducing oxidative stress in plant cells. Such adverse effects result in damage to cell membranes, reduced metabolic activity, and disturbances in photosynthesis, compromising growth [5,8]. However, some aluminum-tolerant plant species prevent its absorption by roots by exuding organic acids into the rhizosphere, and other species accumulate aluminum in leaves and roots without toxicity symptoms [37].

High aluminum levels mainly affect the roots, leading to the growth attenuation of both primary and side roots, which consequently impairs the growth of the shoot [38,39]. In *C. juncea*, there was a reduction in the dry mass length of the roots in soil with aluminum, which results in a smaller contact area between the root and the soil, leading to reduced absorption of nutrients and water. This can be attributed to several factors, such as root regulatory proteins, ferroxidases of the phosphate deficiency signaling pathway, and reactive oxygen species [40,41]. 

In soils with available aluminum, *C. juncea* presented a higher mean of internal carbon, demonstrating an indirect relationship between the aluminum availability and the internal carbon assimilation by the plant, suggesting a compromise in the activity of Ribulose-1-5-bisphosphate carboxylase oxidase (RubisCO), a molecule essential for carbon fixation in photosynthesis in plants exposed to aluminum [39,42]. The internal carbon output is one of the mechanisms that estimate the understanding of the relationship between carbon fixation and water loss. However, it can present challenges for its effective interpretation, as stomatal opening and closing can vary according to the abiotic stimulus, and another factor is leaf variability between species [43,44].

The structure of leaf mesophyll, including aspects such as thickness, the area in contact with intercellular spaces, and cellular density, plays a crucial role in plant photosynthetic efficiency [45,46]. Under variable environmental conditions, these characteristics can be altered, directly influencing plants’ ability to assimilate carbon [47,48,49]. Moreover, studies indicate that mesophyll permeability may be a significant limitation for photosynthesis due to anatomical adaptations that restrict CO_2_ diffusion through mesophyll intercellular spaces [50]. These morpho-anatomical adaptations are essential to understanding limitations in plant photosynthetic processes under abiotic stress conditions [50,51].

Photosynthesis in plants is strongly influenced by stomatal conductance, which has an intrinsic relationship with photosynthetic rates. Stomata play a crucial role in adjusting stomatal conductance in response to environmental conditions, balancing the CO_2_ uptake required for photosynthesis with water loss through transpiration to maintain stomatal conductance regulation and photosynthetic efficiency [52,53,54,55]. Therefore, the increase in stomatal conductance, together with the reduction in photosynthesis and the efficiency of water use in *C. juncea* indicates an imbalance in the opening and closing processes of stomata. Stomatal conductance is influenced by several factors, including aluminum stress. 

Research on plants such as wheat (*Triticum aestivum* L.) and soybean (*Glycine max*) reveals that aluminum can decrease, or in certain cases, increase stomatal conductance, especially when combined with elevated levels of CO_2_ and salt stress [7,26,54,56]. In soybeans, low aluminum concentrations (0.2; 0.4; 0.6; 0.8 g.kg soil^−1^) can be beneficial, while higher concentrations reduce photosynthesis and alter physiological characteristics, demonstrating a complex relationship between aluminum and stomatal conductance [57].

It is suggested that *C. juncea* can use the increase in amino acid synthesis in both roots and leaves as a defense mechanism since organic acids act as precursors in the biosynthesis of these compounds [8,58]. Plants can accumulate osmotically active substances, including amino acids, to mitigate adverse environmental impacts. Among these molecules, proline can assist in osmotic adjustment and maintaining membrane stability [59].

The presence of aluminum in the soil has influenced the increase in the transpiration of *C. juncea* external environment, which facilitates water loss; this relationship is important for water balance [60]. This result may have contributed to a compensation effect for low root growth since the length of the roots, which consequently affects water absorption, is possibly related to the variation between the shoot and root fresh mass. 

The impact on pigment content in species *C. juncea* and *C. spectabilis* demonstrated a decrease in the content of chlorophyll a and carotenoids and an increase in chlorophyll b, which suggests possible damage to the photosynthetic process, and to compensate for this damage, there was an increase in the chlorophyll b content. Based on our results, we can deduce that the reduction in the photosynthetic performance of the genotypes can be attributed to the damage in pigments [61] The presence of Al can cause a deficiency in the production of chlorophyll, as aluminum competes with magnesium, essential for the chlorophyll molecule, for binding sites in the plasma membrane of the roots; this interferes with the absorption and transport of magnesium, affecting the chlorophyll synthesis levels [62]. Other possibilities could be due to the decline in the catalytic activity of RubisCO or the inhibition of chlorophyll synthesis by the decrease in Fe^2+^ ions [63,64].

### 4.2. Nodulation and Translocation of Nitrogenous Compounds in Plants 

Some legume species have the advantage of developing independently of nitrogen fertilizers due to their ability to establish a symbiotic relationship with bacteria. These organisms have the ability to fix atmospheric di-nitrogen within infected cells in root nodules [65]. Symbiosis is known to promote plant growth [66], thus playing a crucial role in acclimating plants to stressful conditions. Through this process, bacteria produce total ureides (allantoin and allantoic acid) that are transported through the xylem, and ammonium is made available to the plant through the host cell (plant) reduction process, resulting in the synthesis of amino acids through GS/GOGAT via [67,68].

*C. juncea* showed a significant increase in both the number of nodules and the number of total ureides and allantoin in the nodules when grown in soil with the presence of Al; Al may possibly have a stimulating effect on the process of biological nitrogen fixation in this species. The importance of ureide degradation goes beyond its function of recycling the nitrogen present in purines, including their potential as protective agents against reactive oxygen species (ROS) [69,70]. This activation results in the accumulation of allantoin and allantoate, substances that help mitigate the negative effects of stress, giving plants greater resistance to adverse conditions [71,72].

On the other hand, the other *Crotalaria* genotypes studied did not demonstrate a significant influence on the production of nitrogen compounds in soil with aluminum, even though *C. spectabilis* presented a greater number of nodules in this condition compared to the limed soil. The interaction between soil pH and nodulation, as well as biological nitrogen fixation, is important since low pH can affect both the bacteria sensitivity and the functioning of nodules; after their establishment, one of the processes affected by pH is the synthesis of nitrogenase in nodules [73,74].

Biological N₂ fixation in legumes, mediated by rhizobia in root nodules, is limited by the availability of phosphorus (P) in the soil, whose bioavailability is often reduced by mineral complexes, especially in acidic soils [75] (Gordon et al., 2001). P deficiency severely affects the metabolic processes in nodules, which are essential for N₂ fixation and its conversion into organic N [12,76,77]. Even under limitation, nodules prioritize the use of P; however, prolonged deprivation reduces Pi levels, impacting the availability of ADP and ATP [12,76].

Plants absorb P as H_2_PO_4_^−^ and HPO_4_^2−^ at pH 4.5–5.8 [78,79], and those adapted to P-poor soils increase acquisition and recycling or improve use efficiency [80,81]. Responses include rhizosphere acidification, the alteration of root architecture, and the expression of Pi transporters [82]. Pi deficiency can reduce photosynthesis due to the energy requirements in the Calvin cycle [83,84].

The increase in protein content in roots and leaves of *C. spectabilis* in soils with available aluminum suggests the possibility of chronic cytotoxicity in plants. This occurs due to the interaction of aluminum with the positively charged layer of the plasma membrane, leading to increased anion transport through plasma membrane transport proteins in proportion to the charge transported by these ions [85].

In the cell plasma membrane, there are transport channels activated by the presence of aluminum [86,87]. These channels are essential for plant resistance to aluminum, as they bind to Al present in the rhizosphere, preventing its entry into the plant [88,89]. Although we have focused on the accumulation of compounds from nitrogen metabolism, the quantification of organic acids may be an alternative to substantiate the strategies adopted by species in response to toxic Al^3+^. It is evident that the synthesis of amino acids in *Crotalaria* species is a fundamental part of the process involved in the exclusion of aluminum, therefore opening up a range of possibilities for understanding tolerance mechanisms in legumes.

### 4.3. Aluminum Tolerance and Accumulation in Plant Biomass

The roots, as they maintain direct contact with the soil, tend to accumulate high concentrations of aluminum (Al), explaining the high levels of this metal in the root biomass. This direct contact facilitates the efficient absorption of Al by the roots, resulting in a greater accumulation of this element in the root cells compared to the plant shoot. The accumulation of aluminum in the roots may be associated with the inhibition of root growth and gas exchange in *C. juncea*, as it faces limitations in the water absorption process due to the lack of root growth. Aluminum has a strong propensity to bind to the root cell wall, with most of the metal remaining in this structure [90]. A study carried out with corn (*Zea mays* L.) showed that aluminum was absorbed and accumulated in the caul [91]. Similarly, in common bean (*Phaseolus vulgaris* L.), aluminum was absorbed and negatively impacted the root elongation zone [92].

Despite being harmful to the roots of most plants and showing low translocation to the shoots, some species are able to accumulate Al in the leaves in concentrations greater than 1000 mg/kg and are called Al accumulators [93]. The translocation of Al from roots to leaves occurs through the xylem, where Al complexes with citrate, malate, oxalate, and fluoride, molecules known for their high binding affinity with Al [94,95]. Usually, species capable of accumulating Al in the shoot are woody plants from tropical regions [96], such as the tea plant (*Camellia sinensis*). 

In soil with aluminum availability, *C. juncea* showed a high concentration of internal carbon, stomatal conductance, and leaf transpiration when comparing different soil conditions, suggesting acclimatization to this environment. In relation to pigments, it stood out with higher levels of chlorophyll b. In terms of biometrics, there was greater length and fresh mass of the shoot and fresh mass of the root in soil with liming, while the shoot dry mass obtained the highest mass in soil with aluminum. In the analysis of nitrogenous compounds, higher amounts of ureides, allantoin, allantoic acid, and amino acids were produced in nodules in soil with aluminum. The roots and leaves showed a higher concentration of allantoic acid and amino acids in soil with aluminum.

*C. spectabilis* presented a higher concentration of chlorophyll b and number of nodules and, regarding nitrogenous compounds in the nodules, it presented a higher concentration of amino acids, as well as a greater production of proteins in the root and leaf under soil conditions with aluminum; however, in limed soil, it showed a higher concentration of allantoic acid in the leaves.

*C. ochroleuca,* when gas exchange and biometry data were evaluated, obtained a lower concentration of chlorophyll b, transpiration, and number of nodules in soil with liming; and in the other condition of soil with aluminum, it showed a high concentration of carotenoids, as well as an increase in the dry mass of the shoot. The nodules showed a higher concentration of allantoic acid in soil with liming, while the amino acids in the nodules showed a higher concentration in soil with aluminum. In leaves, the concentration of amino acids was higher in limed soil.

Expanding the practical applications of *Crotalaria* species in agricultural contexts shows promise due to their ability to accumulate toxic metals and improve soil quality. *C. juncea*, for instance, is recognized for nutrient recycling and its capacity to accumulate potentially toxic metals like lead, nickel, and cadmium. These plants are ideal for soil remediation and the restoration of degraded areas. Moreover, as legumes, all *Crotalaria* species, including *C. juncea*, play a crucial role in green manuring by fixing nitrogen and reducing the need for nitrogen-based fertilizers, thereby promoting more sustainable agricultural practices. In acidic soils, such as those in the Cerrado, the aluminum tolerance of species like *C. ochroleuca* and *C. spectabilis* can sustain agricultural productivity without heavy liming. Additionally, the high biomass production of these plants can be utilized for bioenergy.

However, the study has limitations, such as focusing on specific phenological stages and conducting experiments under controlled conditions like greenhouse settings, which may not fully reflect field conditions. Most studies have evaluated species responses during vegetative phenological stages, leaving long-term effects and interactions with other soil elements understudied.

For future research, it is recommended to explore the complete life cycle of the studied species, conduct field experiments, and investigate the physiological and molecular mechanisms of aluminum tolerance. It is also crucial to study interactions between aluminum and other soil elements and assess the environmental and economic impact of *Crotalaria* use in agricultural systems. These investigations will contribute to more efficient and sustainable agricultural practices.

## 5. Conclusions 

*Crotalaria* species are tolerant to aluminum present in the soil. This capacity is mainly related to the accumulation of aluminum in the roots, which, although resulting in damage to root biomass, indicates an adaptation mechanism. In *C. juncea*, the accumulation of aluminum in the roots affects water absorption and gas exchange, but the species is still able to maintain biological nitrogen fixation, demonstrating a remarkable resistance to aluminum. On the other hand, *C. spectabilis* and *C. ochroleuca* show few physiological changes, which suggests a lower accumulation of aluminum in biomass and a less significant impact on metabolism. Despite the advantages, it is crucial to conduct further research to understand the long-term effects of aluminum exposure, interactions with other soil elements, and the molecular mechanisms underlying plant tolerance. Field studies and studying the reproductive phenological stage are necessary to develop sustainable agricultural practices that maximize the potential of Crotalaria species in various agricultural environments. However, these characteristics highlight the resilience of *Crotalaria* species in environments with high aluminum concentrations, highlighting a complex interaction between aluminum accumulation and the physiological adaptation of these plants.

## Figures and Tables

**Table 1:** Chemical and physical characterization of the soil before cultivation, initial soil, and initial soil with fertilization and liming.

Chemical Characterization of the Soil			
With Fertilization			Treatments
With Al^3+^	Without Al^3+^	Initial	
4.5	5.0	4.33	pH
32	49	12	V%
5	1	11.0	Al^3^ (mmol_c_/dm^3^)
10.4	17.1	4.4	Sum of bases de bases (mmol_c_/dm^3^)
6	9	3.0	Ca^2+^ (mmol_c_/dm^3^)
1.4	1.1	0.4	K^+^ (mmol_c_/dm^3^)
3	7	1.00	Mg^2+^ (mmol_c_/dm^3^)
22	18	31.50	Potential acidity (H^+^ + Al^3+^) (mmol_c_/dm^3^)
7	7	7.0	Organic matter (g/dm^3^)
32.4	35.1	35.4	Cation exchange capacity-CTC (mmol_c_/dm^3^)
3	1	1.0	P-resin (mg/dm^3^)

**Table 2:** Physical characterization of the soil before cultivation, initial soil, and initial soil with fertilization and liming.

Physical Characterization of the Soil (g/Kg)			
With Fertilization			Treatments
Without Al ^3+^	Whit Al^3+^	Initial	
490	487	451	Clay
390	395	375	Sand
120	118	174	Silt

## Data Availability

Data are contained within the article and Supplementary Materials.

## Supplementary Materials

The following supporting information can be downloaded at: https://www.mdpi.com/article/10.3390/plants13162292/s1, Table S1. Table of analysis of variance of total chlorophyll. Degrees of freedom (GL), Sum of squares (SQ), Mean square (QM), F value (Fc), Probability associated with the F value (Pr > Fc). Species represent the three species of Crotalaria (*C. juncea*, *C. ochroleuca*, and *C. spectabilis*), and Soil represents the two soil conditions (with available aluminum and with liming); Table S2. Table of analysis of variance for net photosynthesis. Degrees of freedom (GL), Sum of squares (SQ), Mean square (QM), F value (Fc), Probability associated with the F value (Pr > Fc). Species represent the three species of Crotalaria (*C. juncea*, *C. ochroleuca*, and *C. spectabilis*), and Soil represents the two soil conditions (with available aluminum and with liming); Table S3. Table of analysis of variance of nodule mass. Degrees of freedom (GL), Sum of squares (SQ), Mean square (QM), F value (Fc), Probability associated with the F value (Pr > Fc). Species represent the three species of Crotalaria (*C. juncea*, *C. ochroleuca*, and *C. spectabilis*), and Soil represents the two soil conditions (with available aluminum and with liming); Table S4. Table of analysis of variance of dry mass of root. Degrees of freedom (GL), Sum of squares (SQ), Mean square (QM), F value (Fc), Probability associated with the F value (Pr > Fc). Species represent the three species of Crotalaria (*C. juncea*, *C. ochroleuca*, and *C. spectabilis*), and Soil represents the two soil conditions (with available aluminum and with liming); Table S5. Table of analysis of variance of ureids in the root. Degrees of freedom (GL), Sum of squares (SQ), Mean square (QM), F value (Fc), Probability associated with the F value (Pr > Fc). Species represent the three species of Crotalaria (*C. juncea*, *C. ochroleuca*, and *C. spectabilis*), and Soil represents the two soil conditions (with available aluminum and with liming); Table S6. Table of analysis of variance of allantoin in the root. Degrees of freedom (GL), Sum of squares (SQ), Mean square (QM), F value (Fc), Probability associated with the F value (Pr > Fc). Species represent the three species of Crotalaria (*C. juncea*, *C. ochroleuca*, and *C. spectabilis*), and Soil represents the two soil conditions (with available aluminum and with liming); Table S7. Table of analysis of variance of ureids in the leave. Degrees of freedom (GL), Sum of squares (SQ), Mean square (QM), F value (Fc), Probability associated with the F value (Pr > Fc). Species represent the three species of Crotalaria (*C. juncea*, *C. ochroleuca*, and *C. spectabilis*), and Soil represents the two soil conditions (with available aluminum and with liming); Table S8. Table of analysis of variance of allantoin in the leave. Degrees of freedom (GL), Sum of squares (SQ), Mean square (QM), F value (Fc), Probability associated with the F value (Pr > Fc). Species represent the three species of Crotalaria (*C. juncea*, *C. ochroleuca*, and *C. spectabilis*), and Soil represents the two soil conditions (with available aluminum and with liming).

## References

[1] Yamamoto Y. (2019). Aluminum Toxicity in Plant Cells: Mechanisms of Cell Death and Inhibition of Cell Elongation. Soil Sci. Plant Nutr..

[2] Shetty R., Vidya C.S.-N., Prakash N.B., Lux A., Vaculík M. (2021). Aluminum Toxicity in Plants and Its Possible Mitigation in Acid Soils by Biochar: A Review. Sci. Total Environ..

[3] Rahman M.A., Lee S.-H., Ji H.C., Kabir A.H., Jones C.S., Lee K.-W. (2018). Importance of Mineral Nutrition for Mitigating Aluminum Toxicity in Plants on Acidic Soils: Current Status and Opportunities. Int. J. Mol. Sci..

[4] Yang J., Fan W., Zheng S. (2019). Mechanisms and Regulation of Aluminum-Induced Secretion of Organic Acid Anions from Plant Roots. J. Zhejiang Univ.-Sci. B.

[5] Panda S.K., Baluška F., Matsumoto H. (2009). Aluminum Stress Signaling in Plants. Plant Signal. Behav..

[6] Yan L., Riaz M., Liu J., Yu M., Cuncang J. (2022). The Aluminum Tolerance and Detoxification Mechanisms in Plants; Recent Advances and Prospects. Crit. Rev. Environ. Sci. Technol..

[7] Zhu C.Q., Zhang J.H., Sun L.M., Zhu L.F., Abliz B., Hu W.J., Zhong C., Bai Z.G., Sajid H., Cao X.C. (2018). Hydrogen Sulfide Alleviates Aluminum Toxicity via Decreasing Apoplast and Symplast Al Contents in Rice. Front. Plant Sci..

[8] Kochian L.V., Piñeros M.A., Liu J., Magalhaes J.V. (2015). Plant Adaptation to Acid Soils: The Molecular Basis for Crop Aluminum Resistance. Annu. Rev. Plant Biol..

[9] Wander A.E., da Cunha C.A. (2016). Locais de concentração de atividades agropecuárias na região centro-oeste. Rev. Tecnol. E Soc..

[10] Martirosyan G.S., Sarikyan K.M., Adjemyan G.J., Hakobyan A.H., Pahlevanyan A.M. (2023). The Influence of Green Manure Crops on the Growth, Development, Yield and Fruits Quality of Eggplant. IOP Conf. Ser. Earth Environ. Sci..

[11] Gut G.A.P., Neto J.V.E., Santos R.D.S., Melo R.F.D., Nogueira D.M., Difante G.D.S., Gurgel A.L.C., Santana Í.L.O. (2022). Intercrops of Grass with Legumes as Green Manure for Agroecological Systems. Aust. J. Crop Sci..

[12] Le Roux M., Wyk B.-E. (2013). A Taxonomic Revision of Amphitrichae, a New Section of Crotalaria (Fabaceae). Syst. Bot..

[13] Yaradua S., Alzahrani D., Bello A. (1453). Phylogenetic Position of West African Species of the *Genus Crotalaria* L. (Crotalarieae, Fabaceae) Based on the Current Infrageneric Classification. Pak. J. Bot..

[14] Silva T., Macêdo G., Soares N., Fonseca M., Lacerda G., Veloso M.D.D., Reis A., Pimenta M., Arrudas S. (2021). Phytoremediation Potential of Crotalaria Juncea Plants in Lead-Contaminated Soils. J. Agric. Sci..

[15] Cardoso P.F., Gratão P.L., Gomes-Junior R.A., Medici L.O., Azevedo R.A. (2005). Response of Crotalaria Juncea to Nickel Exposure. Braz. J. Plant Physiol..

[16] Uraguchi S., Watanabe I., Yoshitomi A., Kiyono M., Kuno K. (2006). Characteristics of Cadmium Accumulation and Tolerance in Novel Cd-Accumulating Crops, Avena Strigosa and Crotalaria Juncea. J. Exp. Bot..

[17] Franke I.L., de Souza Marinho J.T. (2020). Adubação verde com Crotalária juncea no cultivo do milho e pastagem em sistema de integração lavoura pecuária na agricultura familiar no Acre. Cad. Agroecol..

[18] Lindino C.A., Tomczak A.P., Gonçalves Junior A.C. (2012). Soil phytoremediation using Crotalaria spectabilis for removal of cadmium and lead. Sci. Agrar. Parana..

[19] Meda A.R., Furlani P.R. (2005). Tolerance to Aluminum Toxicity by Tropical Leguminous Plants Used as Cover Crops. Braz. Arch. Biol. Technol..

[20] dos Santos H.G., Jacomine P.K.T., dos Anjos L.H.C., de Oliveira V.A., Lumbreras J.F., Coelho M.R., de Almeida J.A., de Araujo Filho J.C., de Oliveira J.B., Cunha T.J.F. (2018). Sistema Brasileiro de Classificação de Solos.

[21] van RAIJ B., de ANDRADE J.C., Cantarella H., Quaggio J.A. (2001). ANÁLISE QUÍMICA PARA AVALIAÇÃO DA FERTILIDADE DE SOLOS TROPICAIS. 300.

[22] Teixeira P.C., Donagemma G.K., Fontana A. (2017). Manual de métodos de análise de solo..

[23] de Sousa D.M.G., Lobato E. (2004). Cerrado: Correção do solo e adubação.

[24] Prado C.H.B.A., Casali C. (2006). Fisiologia Vegetal: Práticas Em Relações Hídricas, Fotossíntese e Nutrição Mineral.

[25] Bomfim N.C.P., Aguilar J.V., Ferreira T.C., dos Santos B.S., de Paiva W.d.S., de Souza L.A., Camargos L.S. (2023). Root Development in Leucaena Leucocephala (Lam.) de Wit Enhances Copper Accumulation. Environ. Sci. Pollut. Res..

[26] Zhang L., Wu X.-X., Wang J., Qi C., Wang X., Wang G., Li M., Li X., Guo Y.-D. (2018). BoALMT1, an Al-Induced Malate Transporter in Cabbage, Enhances Aluminum Tolerance in Arabidopsis Thaliana. Front. Plant Sci..

[27] Parkinson K.J., DAY W., Leach J.E. (1980). A Portable System for Measuring the Photosynthesis and Transpiration of Graminaceous Leaves. J. Exp. Bot..

[28] Marshall B., Biscoe P.V. (1980). A Model for C3 Leaves Describing the Dependence of Net Photosynthesis on Irradiance. J. Exp. Bot..

[29] Hiscox J.D., Israelstam G.F. (1979). A method for the extraction of chlorophyll from leaf tissue without maceration. Can. J. Bot..

[30] Wellburn A.R. (1994). The Spectral Determination of Chlorophylls a and b, as Well as Total Carotenoids, Using Various Solvents with Spectrophotometers of Different Resolution. J. Plant Physiol..

[31] Bieleski R.L., Turner N.A. (1966). Separation and Estimation of Amino Acids in Crude Plant Extracts by Thin-Layer Electrophoresis and Chromatography. Anal. Biochem..

[32] Yemm E.W., Cocking E.C., Ricketts R.E. (1955). The Determination of Amino-Acids with Ninhydrin. Analyst.

[33] Vogels G.D., Van Der Drift C. (1970). Differential Analyses of Glyoxylate Derivatives. Anal. Biochem..

[34] Bradford M.M. (1976). A Rapid and Sensitive Method for the Quantitation of Microgram Quantities of Protein Utilizing the Principle of Protein-Dye Binding. Anal. Biochem..

[35] Malavolta E., Vitti G.C., Oliveira S.A. (1997). de Avaliação do Estado Nutricional das Plantas: Princípios e Aplicações..

[36] R: The R Project for Statistical Computing. https://www.r-project.org/.

[37] Bressan A.C.G., de Oliveira Carvalho Bittencourt B.M., Silva G.S., Habermann G. (2021). Could the Absence of Aluminum (Al) Impair the Development of an Al-Accumulating Woody Species from Brazilian Savanna?. Theor. Exp. Plant Physiol..

[38] Banhos O.F.A.A., de O. Carvalho B.M., da Veiga E.B., Bressan A.C.G., Tanaka F.A.O., Habermann G. (2016). A Diminuição Da Assimilação de CO_2_ Induzida Pelo Alumínio No Limoeiro “Cravo” Está Associada à Baixa Condutância Estomática e Não Ao Baixo Desempenho Fotoquímico. Sci. Hortic..

[39] Silva C.M.S., Zhang C., Habermann G., Delhaize E., Ryan P.R. (2018). Does the Major Aluminium-Resistance Gene in Wheat, TaALMT1, Also Confer Tolerance to Alkaline Soils?. Plant Soil.

[40] Dong Z., Shi L., Wang Y., Chen L., Cai Z., Wang Y., Jin J., Li X. (2013). Identification and Dynamic Regulation of microRNAs Involved in Salt Stress Responses in Functional Soybean Nodules by High-Throughput Sequencing. Int. J. Mol. Sci..

[41] Wang X., Wang Z., Zheng Z., Dong J., Song L., Sui L., Nussaume L., Desnos T., Liu D. (2019). Genetic Dissection of Fe-Dependent Signaling in Root Developmental Responses to Phosphate Deficiency. Plant Physiol..

[42] Silva S., Pinto G., Dias M.C., Correia C.M., Moutinho-Pereira J., Pinto-Carnide O., Santos C. (2012). Aluminium Long-Term Stress Differently Affects Photosynthesis in Rye Genotypes. Plant Physiol. Biochem..

[43] Busch F.A., Ainsworth E.A., Amtmann A., Cavanagh A.P., Driever S.M., Ferguson J.N., Kromdijk J., Lawson T., Leakey A.D.B., Matthews J.S.A. (2024). A Guide to Photosynthetic Gas Exchange Measurements: Fundamental Principles, Best Practice and Potential Pitfalls. Plant Cell Environ..

[44] Tominaga J., Shimada H., Kawamitsu Y. (2018). Direct Measurement of Intercellular CO_2_ Concentration in a Gas-Exchange System Resolves Overestimation Using the Standard Method. J. Exp. Bot..

[45] Tomás M., Flexas J., Copolovici L., Galmés J., Hallik L., Medrano H., Ribas-Carbó M., Tosens T., Vislap V., Niinemets Ü. (2013). Importance of Leaf Anatomy in Determining Mesophyll Diffusion Conductance to CO_2_ across Species: Quantitative Limitations and Scaling up by Models. J. Exp. Bot..

[46] Peguero-Pina J.J., Flexas J., Galmés J., Niinemets U., Sancho-Knapik D., Barredo G., Villarroya D., Gil-Pelegrín E. (2012). Leaf Anatomical Properties in Relation to Differences in Mesophyll Conductance to CO_2_ and Photosynthesis in Two Related Mediterranean Abies Species. Plant Cell Environ..

[47] Evans J.R., Kaldenhoff R., Genty B., Terashima I. (2009). Resistances along the CO_2_ Diffusion Pathway inside Leaves. J. Exp. Bot..

[48] Hanba Y., Miyazawa S.-I., Kogami H., Terashima I. (2001). Effects of Leaf Age on Internal CO_2_ Transfer Conductance and Photosynthesis in Tree Species Having Different Types of Shoot Phenology. Funct. Plant Biol..

[49] Terashima I., Hanba Y.T., Tholen D., Niinemets Ü. (2011). Leaf Functional Anatomy in Relation to Photosynthesis. Plant Physiol..

[50] Sáez P.L., Bravo L.A., Cavieres L.A., Vallejos V., Sanhueza C., Font-Carrascosa M., Gil-Pelegrín E., Javier Peguero-Pina J., Galmés J. (2017). Photosynthetic Limitations in Two Antarctic Vascular Plants: Importance of Leaf Anatomical Traits and Rubisco Kinetic Parameters. J. Exp. Bot..

[51] Galmés J., Medrano H., Flexas J. (2007). Photosynthetic limitations in response to water stress and recovery in Mediterranean plants with different growth forms. New Phytol..

[52] Baroli I., Price G.D., Badger M.R., von Caemmerer S. (2008). The Contribution of Photosynthesis to the Red Light Response of Stomatal Conductance. Plant Physiol..

[53] Lawson T., von Caemmerer S., Baroli I., Lüttge U.E., Beyschlag W., Büdel B., Francis D. (2011). Photosynthesis and Stomatal Behaviour. Progress in Botany 72.

[54] Overdieck D., Overdieck D. (2016). Water Use Efficiency and Stomatal Conductance. CO_2_, Temperature, and Trees: Experimental Approaches.

[55] Tuzet A., Perrier A., Leuning R. (2003). A Coupled Model of Stomatal Conductance, Photosynthesis and Transpiration. Plant Cell Environ..

[56] Ridolfi M., Garrec J.-P. (2000). Consequences of an Excess Al and a Deficiency in Ca and Mg for Stomatal Functioning and Net Carbon Assimilation of Beech Leaves. Ann. For. Sci..

[57] Zhang X.-B., Liu P., Yang Y.S., Xu G.-D. (2007). Effect of Al in Soil on Photosynthesis and Related Morphological and Physiological Characteristics of Two Soybean Genotypes. Bot. Stud..

[58] López-Bucio J., Nieto-Jacobo M.F., Ramírez-Rodríguez V., Herrera-Estrella L. (2000). Organic Acid Metabolism in Plants: From Adaptive Physiology to Transgenic Varieties for Cultivation in Extreme Soils. Plant Sci..

[59] Raza A., Charagh S., Abbas S., Hassan M.U., Saeed F., Haider S., Sharif R., Anand A., Corpas F.J., Jin W. (2023). Assessment of Proline Function in Higher Plants under Extreme Temperatures. Plant Biol..

[60] Taiz L., Zeiger E., Møller I.M., Murphy A. (2021). Fundamentos de Fisiologia Vegetal.

[61] Sharma V., Yadav M., Kumari N. (2018). Aluminium Fluoride Induced Changes in Chlorophyll a Fluorescence, Antioxidants and Psb A Gene Expression of Brassica Juncea Cultivars. J. Plant Interact..

[62] Ali B., Hasan S.A., Hayat S., Hayat Q., Yadav S., Fariduddin Q., Ahmad A. (2008). Um Papel Dos Brassinosteróides Na Melhoria Do Estresse Do Alumínio Por Meio Do Sistema Antioxidante No Feijão Mungo ( *Vigna* Radiata L. Wilczek). Environ. Exp. Bot..

[63] Batra N.G., Sharma V., Kumari N. (2014). Drought-Induced Changes in Chlorophyll Fluorescence, Photosynthetic Pigments, and Thylakoid Membrane Proteins of Vigna Radiata. J. Plant Interact..

[64] Ram A., Verma P., Gadi B. (2014). Effect of Fluoride and Salicylic Acid on Seedling Growth and Biochemical Parameters of Watermelon (Citrullus Lanatus). Fluoride.

[65] Lu M.-Z., Carter A.M., Tegeder M. (2022). Altering Ureide Transport in Nodulated Soybean Results in Whole-Plant Adjustments of Metabolism, Assimilate Partitioning, and Sink Strength. J. Plant Physiol..

[66] Glick B.R. (1995). The Enhancement of Plant Growth by Free-Living Bacteria. Can. J. Microbiol..

[67] Atkins C.A., Smith P.M.C. (2007). Translocation in Legumes: Assimilates, Nutrients, and Signaling Molecules. Plant Physiol..

[68] Ferreira T.C., Aguilar J.V., Souza L.A., Justino G.C., Aguiar L.F., Camargos L.S. (2016). pH Effects on Nodulation and Biological Nitrogen Fixation in Calopogonium Mucunoides. Braz. J. Bot..

[69] Brychkova G., Alikulov Z., Fluhr R., Sagi M. (2008). A Critical Role for Ureides in Dark and Senescence-Induced Purine Remobilization Is Unmasked in the Atxdh1 Arabidopsis Mutant. Plant J..

[70] Takagi H., Ishiga Y., Watanabe S., Konishi T., Egusa M., Akiyoshi N., Matsuura T., Mori I.C., Hirayama T., Kaminaka H. (2016). Allantoin, a Stress-Related Purine Metabolite, Can Activate Jasmonate Signaling in a MYC2-Regulated and Abscisic Acid-Dependent Manner. J. Exp. Bot..

[71] Alamillo J.M., Díaz-Leal J.L., Sánchez-Moran M.V., Pineda M. (2010). Molecular Analysis of Ureide Accumulation under Drought Stress in *Phaseolus vulgaris* L. Plant Cell Environ..

[72] Watanabe S., Matsumoto M., Hakomori Y., Takagi H., Shimada H., Sakamoto A. (2014). The Purine Metabolite Allantoin Enhances Abiotic Stress Tolerance through Synergistic Activation of Abscisic Acid Metabolism. Plant Cell Environ..

[73] Bordeleau L.M., Prévost D. (1994). Nodulation and Nitrogen Fixation in Extreme Environments. Plant Soil.

[74] Lin M.-H., Gresshoff P.M., Ferguson B.J. (2012). Systemic Regulation of Soybean Nodulation by Acidic Growth Conditions. Plant Physiol..

[75] Gordon A.J., Lea P.J., Rosenberg C., Trinchant J.-C., Lea P.J., Morot-Gaudry J.-F. (2001). Nodule Formation and Function. Plant Nitrogen.

[76] Plaxton W.C., Podestá F.E. (2006). The Functional Organization and Control of Plant Respiration. Crit. Rev. Plant Sci..

[77] Sulieman S., Ha C.V., Schulze J., Tran L.-S.P. (2013). Growth and Nodulation of Symbiotic Medicago Truncatula at Different Levels of Phosphorus Availability. J. Exp. Bot..

[78] Hinsinger P. (2001). Bioavailability of Soil Inorganic P in the Rhizosphere as Affected by Root-Induced Chemical Changes: A Review. Plant Soil.

[79] Raghothama K.G. (1999). Phosphate acquisition. Annu. Rev. Plant Biol..

[80] Lambers H., Raven J.A., Shaver G.R., Smith S.E. (2008). Plant Nutrient-Acquisition Strategies Change with Soil Age. Trends Ecol. Evol..

[81] Péret B., Clément M., Nussaume L., Desnos T. (2011). Root Developmental Adaptation to Phosphate Starvation: Better Safe than Sorry. Trends Plant Sci..

[82] Vance C.P., Uhde-Stone C., Allan D.L. (2003). Phosphorus Acquisition and Use: Critical Adaptations by Plants for Securing a Nonrenewable Resource. New Phytol..

[83] Fredeen A.L., Raab T.K., Rao I.M., Terry N. (1990). Effects of Phosphorus Nutrition on Photosynthesis in Glycine Max (L.) Merr. Planta.

[84] Hellsten A., Huss-Danell K. (2000). Interaction Effects of Nitrogen and Phosphorus on Nodulation in Red Clover (*Trifolium pratense* L.). Acta Agric. Scand. Sect. B—Soil Plant Sci..

[85] Singh S., Tripathi D.K., Singh S., Sharma S., Dubey N.K., Chauhan D.K., Vaculík M. (2017). Toxicity of Aluminium on Various Levels of Plant Cells and Organism: A Review. Environ. Exp. Bot..

[86] Meyer S., Mumm P., Imes D., Endler A., Weder B., Al-Rasheid K.A.S., Geiger D., Marten I., Martinoia E., Hedrich R. (2010). AtALMT12 Represents an R-Type Anion Channel Required for Stomatal Movement in Arabidopsis Guard Cells. Plant J..

[87] Roelfsema M.R.G., Hedrich R. (2005). In the Light of Stomatal Opening: New Insights into ‘the Watergate’. New Phytol..

[88] Hoekenga O.A., Maron L.G., Piñeros M.A., Cançado G.M.A., Shaff J., Kobayashi Y., Ryan P.R., Dong B., Delhaize E., Sasaki T. (2006). AtALMT1, Which Encodes a Malate Transporter, Is Identified as One of Several Genes Critical for Aluminum Tolerance in Arabidopsis. Proc. Natl. Acad. Sci. USA.

[89] Sasaki T., Yamamoto Y., Ezaki B., Katsuhara M., Ahn S.J., Ryan P.R., Delhaize E., Matsumoto H. (2004). A Wheat Gene Encoding an Aluminum-Activated Malate Transporter. Plant J..

[90] Horst W.J., Wang Y., Eticha D. (2010). The Role of the Root Apoplast in Aluminium-Induced Inhibition of Root Elongation and in Aluminium Resistance of Plants: A Review. Ann. Bot..

[91] Bennet R.J., Breen C.M., Bandu V. (1985). Aluminium Toxicity and Regeneration of the Root Cap: Preliminary Evidence for a Golgi Apparatus Derived Morphogen in the Primary Root of Zea Mays. South Afr. J. Bot..

[92] Rangel A.F., Rao I.M., Horst W.J. (2007). Spatial Aluminium Sensitivity of Root Apices of Two Common Bean (*Phaseolus vulgaris* L.) Genotypes with Contrasting Aluminium Resistance. J. Exp. Bot..

[93] Tolrà R., Vogel-Mikuš K., Hajiboland R., Kump P., Pongrac P., Kaulich B., Gianoncelli A., Babin V., Barceló J., Regvar M. (2011). Localization of Aluminium in Tea (Camellia Sinensis) Leaves Using Low Energy X-Ray Fluorescence Spectro-Microscopy. J. Plant Res..

[94] Vitorello V.A., Capaldi F.R., Stefanuto V.A. (2005). Recent Advances in Aluminum Toxicity and Resistance in Higher Plants. Braz. J. Plant Physiol..

[95] Ma J.F., Ryan P.R., Delhaize E. (2001). Aluminium Tolerance in Plants and the Complexing Role of Organic Acids. Trends Plant Sci..

[96] Jansen S., Broadley M., Robbrecht E., Smets E. (2002). Aluminum Hyperaccumulation in Angiosperms: A Review of Its Phylogenetic Significance. Bot. Rev..

