# Correction: dos Santos et al. Physiological Responses of *Crotalaria* spp. to the Presence of High Aluminum Availability in the Soil. *Plants* 2024, *13*, 2292

**DOI:** 10.3390/plants15101430

**Published:** 2026-05-08

**Authors:** Beatriz Silvério dos Santos, Tassia Caroline Ferreira, Maiara Luzia Grigoli Olívio, Lucas Anjos de Souza, Liliane Santos de Camargos

**Affiliations:** 1 Plant Metabolism Physiology Laboratory, Department of Biology and Zootechny, School of Engineering, São Paulo State University (UNESP), Rua Monção, 226, Zona Norte, Ilha Solteira 15385-000, SP, Brazil; beatriz.silverio-santos@unesp.br (B.S.d.S.); tassia.ferreira@unesp.br (T.C.F.); maiara.olivio@unesp.br (M.L.G.O.); 2 Instituto Federal Goiano, Campus Rio Verde, Rio Verde 75901-970, GO, Brazil; lucas.anjos@ifgoiano.edu.br

## Error in Figure 1

In the original publication [1], there was an inversion of the species names *C. spectabilis* and *C. ochroleuca* in Figure 1, both in the illustrative representation of species morphology and in the figure legend. Specifically, the image labeled as *C. ochroleuca* actually corresponds to *C. spectabilis*. In addition, in the original legend, the treatment assignments for T3–T6 were incorrectly attributed: T3 = *C. ochroleuca* with aluminum, T4 = *C. ochroleuca* with liming, T5 = *C. spectabilis* with aluminum, and T6 = *C. spectabilis* with liming.

The corrected Figure 1 and its revised legend are provided below. The corrected legend reads as follows:

**Figure 1:**
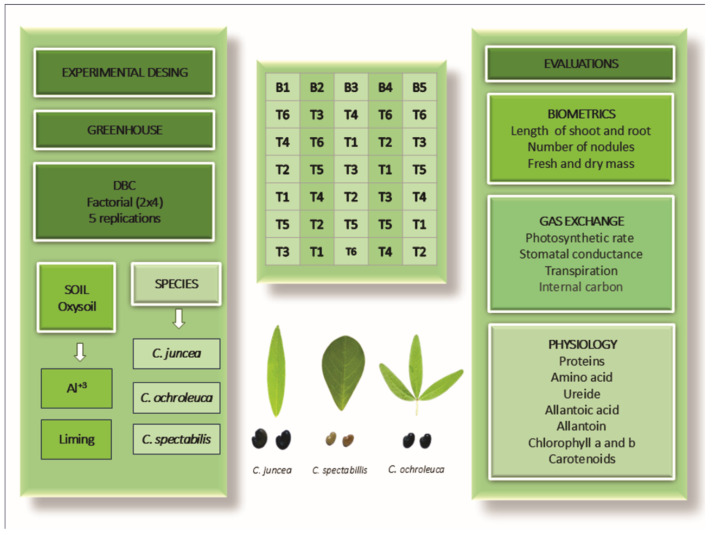
Scheme of design and experimental design in a factorial layout with the distribution of each treatment (Diagram): T1 = *C. juncea* with aluminum, T2 = *C. juncea* with liming, T3 = *C. spectabilis* with aluminum, T4 = *C. spectabilis* with liming, T5 = *C. ochroleuca* with aluminum, and T6 = *C. ochroleuca* with liming and types of evaluations (biometrics, gas exchange, and physiology).

The authors state that the scientific conclusions are unaffected. This correction was approved by the Academic Editor. The original publication has also been updated.
