# Supplementary material for: Physiological Responses of Crotalaria spp. to the Presence of High Aluminum Availability in the Soil
Source: Plants (Basel). 2024 Aug 17;13(16):2292. doi: 10.3390/plants13162292 (PMC11359568; doi:10.3390/plants13162292)
Supplement: Supplementary file 1 [file plants-13-02292-s001.zip › plants-3099886-supplementary.pdf]

SUPPLEMENTARY MATERIAL

Table S1. Table of analysis of variance of total chlorophyll. Degrees of freedom (GL), Sum of squares (SQ), Mean square (QM), F value (Fc), Probability associated with the F value (Pr>Fc). Species represent the three species of Crotalaria (C. juncea, C. ochroleuca, and C. spectabilis), and Soil represents the two soil conditions (with available aluminum and with liming)

Table of analysis of variance of total chlorophyll					
	GL	SQ	QM	Fc	Pr>Fc
Block	4	13.36	5	0.3115	0.86689
Species	2	204.82	3	9.5480	0.00123
Soil	1	29.12	4	2.7148	0.11504
Species * Soil	2	70.84	6	3.3021	0.05765
Residue	20	214.52	2		
Total	29	532.65	1		
CV= 81.01%					

CV: coefficient of variation; *: significant difference at 5%; **: significant difference at 1%; ns: not significant at 5% probability by F Test

Table S2. Table of analysis of variance for net photosynthesis. Degrees of freedom (GL), Sum of squares (SQ), Mean square (QM), F value (Fc), Probability associated with the F value (Pr>Fc). Species represent the three species of Crotalaria (C. juncea, C. ochroleuca, and C. spectabilis), and Soil represents the two soil conditions (with available aluminum and with liming)

Table of analysis of variance of photosynthesis liquidates					
	GL	SQ	QM	Fc	Pr>Fc
Block	4	158.74	4	0.9680	0.44675
Species	2	604.85	3	7.3765	0.00398
Soil	1	1.29	2	2.7148	0.86108
Species * Soil	2	11.40	6	0.1390	0.87102
Residue	20	819.97	5		
Total	29	1596.25	1		
CV= 25.16%					

CV: coefficient of variation; *: significant difference at 5%; **: significant difference at 1%; ns: not significant at 5% probability by F Test

Table S3. Table of analysis of variance of nodule mass. Degrees of freedom (GL), Sum of squares (SQ), Mean square (QM), F value (Fc), Probability associated with the F value (Pr>Fc). Species represent the three species of Crotalaria (C. juncea, C. ochroleuca, and C. spectabilis), and Soil represents the two soil conditions (with available aluminum and with liming)

Table of analysis of variance of nodule mass					
	GL	SQ	QM	Fc	Pr>Fc
Block	4	0.00550	3	0.306	0.87076
Species	2	0.29230	6	32.511	0.00000
Soil	1	0.00048	2	0.107	0.74643

Species * Soil	2	0.01115	5	1.240	0.31064
Residue	20	0.08991	4		
Total	29	0.39934	1		
CV= 32.87%					

CV: coefficient of variation; *: significant difference at 5%; **: significant difference at 1%; ns: not significant at 5% probability by F Test

Table S4. Table of analysis of variance of dry mass of root. Degrees of freedom (GL), Sum of squares (SQ), Mean square (QM), F value (Fc), Probability associated with the F value (Pr>Fc). Species represent the three species of Crotalaria (C. juncea, C. ochroleuca, and C. spectabilis), and Soil represents the two soil conditions (with available aluminum and with liming)

Table of analysis of variance of dry mass of root					
	GL	SQ	QM	Fc	Pr>Fc
Block	4	0.07141	2	0.7570	0.56527
Species	2	1.18725	6	25.1713	0.00000
Soil	1	0.23408	5	9.9258	0.00503
Species * Soil	2	0.12389	4	2.6266	0.09708
Residue	20	0.47167	3		
Total	29	2.08830	1		
CV= 41.54%					

CV: coefficient of variation; *: significant difference at 5%; **: significant difference at 1%; ns: not significant at 5% probability by F Test

Table S5. Table of analysis of variance of ureids in the root. Degrees of freedom (GL), Sum of squares (SQ), Mean square (QM), F value (Fc), Probability associated with the F value (Pr>Fc). Species represent the three species of Crotalaria (C. juncea, C. ochroleuca, and C. spectabilis), and Soil represents the two soil conditions (with available aluminum and with liming)

Table of analysis of variance of ureids in the root					
	GL	SQ	QM	Fc	Pr>Fc
Block	4	2.6998	5	2.3912	0.08500
Species	2	1.3328	4	2.3610	0.12007
Soil	1	3.3433	6	11.8451	0.00258
Species * Soil	2	0.2648	2	0.4692	0.63224
Residue	20	5.6451	3		
Total	29	13.2859	1		
CV= 30.1%					

CV: coefficient of variation; *: significant difference at 5%; **: significant difference at 1%; ns: not significant at 5% probability by F Test

Table S6. Table of analysis of variance of allantoin in the root. Degrees of freedom (GL), Sum of squares (SQ), Mean square (QM), F value (Fc), Probability associated with the F value (Pr>Fc). Species represent the three species of Crotalaria (C. juncea, C. ochroleuca, and C. spectabilis), and Soil represents the two soil conditions (with available aluminum and with liming)

Table of analysis of variance of allantoin in the Root					
	GL	SQ	QM	Fc	Pr>Fc
Block	4	1.3524	3	2.0106	0.131719
Species	2	3.8742	6	11.5189	0.000470
Soil	1	1.1419	5	6.7904	0.016912
Species * Soil	2	0.8232	4	2.4477	0.111973
Residue	20	3.3633	2		
Total	29	10.5551	1		
CV= 34.4%					

CV: coefficient of variation; *: significant difference at 5%; **: significant difference at 1%; ns: not significant at 5% probability by F Test

Table S7. Table of analysis of variance of ureids in the leave. Degrees of freedom (GL), Sum of squares (SQ), Mean square (QM), F value (Fc), Probability associated with the F value (Pr>Fc). Species represent the three species of Crotalaria (C. juncea, C. ochroleuca, and C. spectabilis), and Soil represents the two soil conditions (with available aluminum and with liming)

Table of analysis of variance of ureids in the leave					
	GL	SQ	QM	Fc	Pr>Fc
Block	4	0.1476	2	0.0763	0.98866
Species	2	9.6622	6	9.9846	0.00098
Soil	1	0.3052	3	0.6308	0.43638
Species * Soil	2	2.4501	5	2.5319	0.10467
Residue	20	9.6771	4		
Total	29	22.2422	1		
CV= 9.78%					

CV: coefficient of variation; *: significant difference at 5%; **: significant difference at 1%; ns: not significant at 5% probability by F Test

Table S8. Table of analysis of variance of allantoin in the leave. Degrees of freedom (GL), Sum of squares (SQ), Mean square (QM), F value (Fc), Probability associated with the F value (Pr>Fc). Species represent the three species of Crotalaria (C. juncea, C. ochroleuca, and C. spectabilis), and Soil represents the two soil conditions (with available aluminum and with liming)

Table of analysis of variance of allantoin in the leave					
	GL	SQ	QM	Fc	Pr>Fc
Block	4	2.5846	5	0.0763	0.41487
Species	2	6.1932	6	9.9846	0.01795
Soil	1	0.0746	2	0.6308	0.73349
Species * Soil	2	0.6553	3	2.5319	0.60030
Residue	20	12.5154	4		
Total	29	22.0231	1		
CV= 11.87%					

CV: coefficient of variation; *: significant difference at 5%; **: significant difference at 1%; ns: not significant at 5% probability by F Test
